# The genomic and phylogenetic characterization of lytic *Pseudomonas aeruginosa* PAK bacteriophages BL4, BL6, and BL7 collected from aquatic samples

**DOI:** 10.1128/mra.00954-24

**Published:** 2024-11-04

**Authors:** Fahareen B. Mosharraf, Austen Rowell, Lisa M. Bono

**Affiliations:** 1 Department of Biological Sciences, Texas Tech University, Lubbock, Texas, USA; Queens College Department of Biology, Queens, New York, USA

**Keywords:** bacteriophages, environmental isolates, genomic characterization, phylogenetic analysis

## Abstract

Environmental phages infecting *Pseudomonas aeruginosa* PAK, an opportunistic pathogen, were isolated from playa lakes in Lubbock, TX. We present the genome sequence of three lytic bacteriophages. Upon analysis of sequence similarity, we identified the viruses as a part of an unclassified species within the genus *Pbunavirus* in the *Caudoviricetes* class.

## ANNOUNCEMENT

*Pseudomonas aeruginosa* strain PAK belongs to the laboratory reference strain of *Pseudomonas aeruginosa* that can cause significant disease as a hospital-acquired opportunistic pathogen. PAK is a highly virulent and drug-resistant gram-negative pathogen that mostly flourishes in terrestrial and aquatic environments (1–4). We isolated, sequenced, and bioinformatically characterized three phages that infect PAK from playa lakes in Lubbock, Texas.

Water samples were collected on 11 August 2022, from Lubbock, TX, at a depth of 7–15 cm and found at GPS coordinates 33.566, –101.803 (BL4 and BL6) and 33.511, –102.002 (BL7). Each sample was filtered through 0.45-micron and 0.22-micron syringe filters to remove debris and undesired bacteria, respectively. Furthermore, 400 µL of stationary PAK culture was inoculated with 200 µL of the filtered sample and incubated overnight at 37°C. After centrifugation at 1,786 g for 30 minutes, the culture was filtered again through a 0.22-micron syringe filter. Phages were isolated using the agar-overlay technique on a lawn of PAK (5) and subjected to triple plaque purification. The genomic DNA of the phages was extracted from phage lysates using the Quantabio Extraction DNA Kit. The concentration and quantity of DNA were evaluated using the BioTek Take3 microvolume plate. The extracted genomes were prepared for whole-genome sequencing using an Illumina DNA Prep Tagmentation Kit and sequenced on the Illumina NextSeq 2000 platform using a 300-cycle flow cell kit to generate 200-bp paired-end reads.

Raw Illumina reads were trimmed and assessed for quality using fastp v0.23.4 (6). Host genome contaminants (NCBI GeneBank accession no. CP020659.1) were eliminated using bowtie2 (7). Genome assembly was conducted with SPAdes v3.15.5 (8) utilizing Illumina paired-end short reads. Coverage and depth were determined using SAMtools v1.6 (9) and BEDTools v2.31.0 (10). Quast v2.2.4 (11) was used to obtain the assembly statistics. The quality and completeness of our genome samples were evaluated using CheckV v1.0.1 (12). Viral taxonomic characterization was performed using NCBI BLASTn, matching the most similar sequences in the NCBI nucleotide database. Annotation of bacteriophage genomes was done using PHROGS (Pharokka v1.3.2) (13, 14). To determine the closest relatives of our isolated phages, we used FastANI v1.34 (15) for average nucleotide identity through pairwise genome comparisons. Default parameters were used for all software tools. Electron micrographs were taken using a Hitachi H-7650 TEM located at the TTU CASM facility using a 1% uranyl acetate stain prior to imaging.

**Fig 1:**
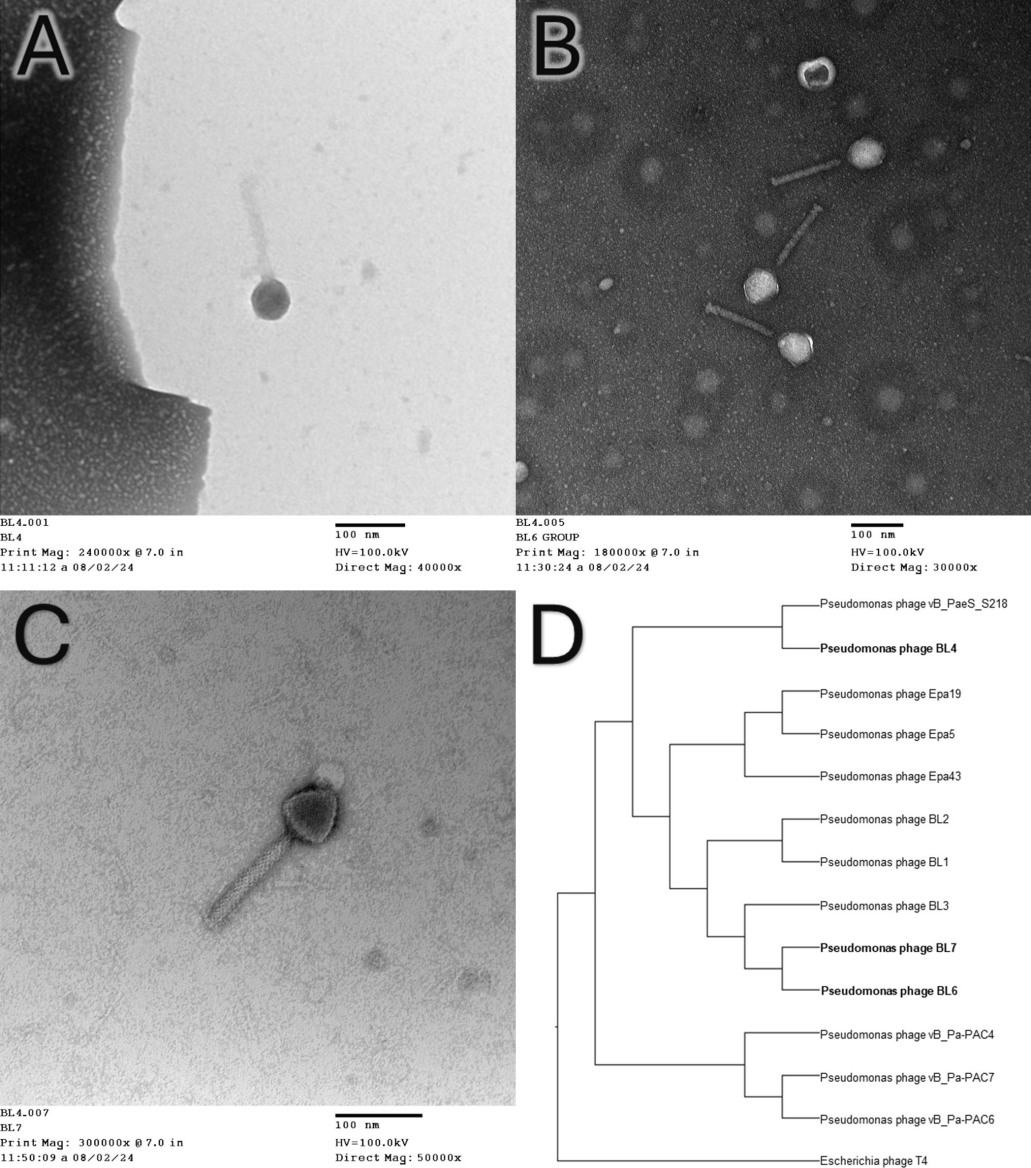
Bacteriophage size and phylogenetic relationship. (**A–C**) Electron micrographs of tailed bacteriophages BL4, BL6, and BL7, respectively. Average length of 130–150 nm and diameter of 30–50 nm. (**D**) Cladogram of newly isolated phage in bold compared to sequences from NCBI nucleotide database.

Our SPAdes assembly metrics confirmed the presence of a single contig exceeding 5,000 base pairs. We were unable to define the start and stop sequences for some coding sequences, which are essential for NCBI’s classification as complete genomes. Consequently, the genome sequences were categorized as partial. Through sequence similarity analysis (refer to Table 1), we determined that the viruses belong to an unclassified species within the genus *Pbunavirus,* classified under the *Caudoviricetes* class. Sizes were determined from the electron micrograph with an average length of 130–150 nm and a head diameter of 30–50 nm (Fig. 1A through C). Sequences from the NCBI nucleotide database, retrieved on 21 June 2024 (16), were aligned using MAFFTv7.526 (17) and then IQ-TREEv2.3.6 (18) to build the phylogenetic tree. Visualization and annotations were completed using FigTreev1.4.4 (19) (Fig. 1D).

**TABLE 1:** Characterization of the reported bacteriophages BL4, BL6, and BL7

Parameter	BL4	BL6	BL7
Genome size (bp)	61,021	66,414	66,029
Total no. of reads generated per sample	4,388,360	1,028,358	4,843,352
Average coverage	1,891	2,755	1,947
CheckV completeness (%)	100%	100%	100%
CheckV quality	High	High	High
GC content (%)	59.97%	61.41%	63.15%
No. of coding sequence	91	1,416	2,360
No. of hypothetical proteins	55	1,305	2,227
No. of tRNAs	0	0	0
Virulence genes	0	0	0
Antibiotic resistance genes	0	0	0
Average nucleotide identity (ANI values in %)	97.29%	97.27%	95.57%
Closest related phage based on average nucleotide identity (GenBank accession no.)	*Pseudomonas* phage Churro OL763419.1	*Pseudomonas* phage Epa39 MT118303.1	*Pseudomonas* phage vB_PaeM_C1-14_Ab28 NC_026600.1
GeneBank accession no.	PP782352	PQ149444	PQ149445
Bioproject accession no.	PRJNA1043698	PRJNA1143577	PRJNA1143578
SRA accession no.	SRP479097	SRR30567586	SRR30567658

## Data Availability

Partial phage genomes were deposited in the GenBank, Bioproject, and NCBI Sequence Read Archive databases (SRA). The GenBank accession numbers are PP782352, PQ149444, and PQ149445, Bioproject numbers are PRJNA1043698, PRJNA1143578, and PRJNA1143578, and the SRA accession numbers are SRP479097, SRR30567586, and SRR30567658.
